# Depression and Its Effect on Geriatric Rehabilitation Outcomes in Switzerland’s Aging Population

**DOI:** 10.3390/medicina61020257

**Published:** 2025-02-02

**Authors:** Bojan Miletic, Antonia Plisic, Lejla Jelovica, Jan Saner, Marcus Hesse, Silvije Segulja, Udo Courteney, Gordana Starcevic-Klasan

**Affiliations:** 1 Department of Geriatrics and Rehabilitation, Lucerne Cantonal Hospital Wolhusen, 6110 Wolhusen, Switzerland; antonia.plisic@student.uniri.hr (A.P.); jan.saner@bluewin.ch (J.S.); udo.courteney@luks.ch (U.C.); 2 Faculty of Health Studies, University of Rijeka, 51000 Rijeka, Croatia; lejla.jelovica@fzsri.uniri.hr (L.J.);

**Keywords:** depression, functional status older adults, outcome, rehabilitation

## Abstract

*Background and Objectives*: Depression is a common mental problem in the older population and has a significant impact on recovery and general well-being. A comprehensive understanding of the prevalence of depressive symptoms and their effects on functional outcomes is essential for improving care strategies. The primary aim of this study was to determine the prevalence of depressive symptoms in older patients undergoing geriatric rehabilitation and to assess their specific impact on their functional abilities. *Materials and Methods*: A retrospective study was conducted at the Lucerne Cantonal Hospital in Wolhusen, Switzerland, spanning from 2015 to 2020 and including 1159 individuals aged 65 years and older. The presence of depressive symptoms was assessed using the Geriatric Depression Scale (GDS) Short Form, while functional abilities were evaluated using the Functional Independence Measure (FIM) and the Tinetti test. Data analysis was performed using TIBCO Statistica 13.3, with statistical significance set at *p* < 0.05. *Results*: Of the participants, 22.9% (N = 266) exhibited depressive symptoms, with no notable differences between genders. Although all patients showed functional improvements, the duration of rehabilitation was prolonged by two days (*p* = 0.012, d = 0.34) in those with depressive symptoms. Alarmingly, 76% of participants were classified as at risk of falling based on the Tinetti score. However, no significant correlation was found between the GDS and Tinetti scores at admission (*p* = 0.835, r = 0.211) or discharge (*p* = 0.336, r = 0.184). The results from the non-parametric Wilcoxon matched-pairs test provide compelling evidence of significant changes in FIM scores when comparing admission scores to those at discharge across all FIM categories. *Conclusions*: Depressive symptoms are particularly common in geriatric rehabilitation patients, leading to prolonged recovery time and increased healthcare costs. While depressive symptoms showed no correlation with mobility impairments, improvements in functional status were directly associated with reduced GDS scores. Considering mental health during admission and planning is critical in optimizing rehabilitation outcomes.

## 1. Introduction

Depression is an immediate problem that is often overlooked in the older population. Alarmingly, almost fifty percent of these cases go undiagnosed, affecting the quality of life of older people [1]. According to the World Health Organization, approximately 10 to 20% of seniors suffer from depressive disorders [2]. Early signs of depression in older adults are often mistakenly attributed to the normal aging process or misinterpreted as other medical problems, such as dementia [3]. Sleep disturbances, whether insomnia or excessive sleep, are usually considered normal age-related changes. Depression can exacerbate the clinical symptoms of chronic diseases and increase the risk of additional health complications, including cardiovascular or cerebrovascular adverse events [4]. It increases the risk of suicide, impairs physical, cognitive, and social functioning, and promotes self-neglect, which in turn leads to increased mortality [5,6]. A study conducted by Siu et al. highlights the alarming deterioration of physical health in people suffering from depression, which often manifests in reduced mobility, lower muscle strength, and an overall decrease in physical resilience [7]. Depression sets in motion a cycle of inactivity and fatigue that accelerates the physical decline. At the same time, insufficient physical activity has a detrimental effect on cardiovascular health, exacerbates chronic diseases, and impairs immune function, making people more susceptible to illness and injury, as the studies by Shao et al. and Ciumărnean et al. show [8,9]. A thorough meta-analysis by Delpino et al. has definitively shown that insufficient physical activity significantly increases the risk of multimorbidity in older adults [10]. Deterioration in physical health not only significantly impairs quality of life, but also represents a significant barrier in overcoming depression, creating a complex, self-repeating cycle [11]. Depression significantly increases the frequency of hospital admissions, and thus represents a critical public health challenge and an urgent socioeconomic problem [12]. Furthermore, depression impairs the ability of older people to live their lives satisfactorily, maintain social contacts, and participate in daily activities [13,14]. Thus, depression significantly worsens the quality of life of older adults, especially when it is accompanied by an increase in comorbidities in this population group, as shown in the studies by Sivertsen et al. and Cavdar et al. [15,16]. Strengthening social ties with family and friends and providing adequate social support from the community in which the older adults live, particularly in the form of educational initiatives targeting both older adults and their support systems, are critical to addressing depression in older adults. These measures can reduce incidence rates and facilitate the rapid identification of depressive symptoms for effective intervention [17,18,19]. Timely detection of depressive symptoms enables the planning and implementation of interventions that can reduce or prevent the negative impact of depression on functional performance and activities of daily living while improving the effectiveness of medical interventions in older adults [20,21]. Geriatric rehabilitation strategies are crucial to promoting health and independence in older people. Essential interventions include exercise programs to promote mobility, strength, and balance, which reduce the risk of falls [22]. Unhjem and co-authors found that maintaining a high muscle strength capacity into old age is associated with positive effects on functional performance that cannot be achieved through leisure activities [23]. This type of exercise is an integral part of the rehabilitation program for older adults, and physical activity is a promising non-pharmaceutical method for the treatment and prevention of depression in this demographic, according to research by Zhang et al. [24]. Cognitive rehabilitation specifically addresses memory and cognitive decline, particularly in people with dementia [25]. Through memory-enhancing games and problem-solving tasks, these strategies effectively slow the cognitive decline and improve mental well-being [26,27]. Occupational therapy seeks to promote self-care by using aids and methods for activities such as dressing-up and meal preparation to improve independence in daily living [28]. This is a crucial element in optimizing functional performance and social participation in older adults, as well as in maintaining independence and promoting safety, as de Coninck et al. found in their study [28]. Nutritional plans are important to combat malnutrition, a pressing problem in the aging population [29,30]. Overall, these interventions are showing impressive results worldwide, leading to greater independence, fewer hospitalizations, and an overall improved quality of life, as several meta-analyses have shown [31,32,33]. Determining the prevalence of depressive symptoms in each locality is essential, as each community has different cultural, geographic, public health, and sociological characteristics that can affect mental health. In this study, the presence of depressive symptoms and their effects on the functional abilities of geriatric rehabilitation patients in Switzerland are examined over six years. A comprehensive retrospective data analysis was conducted to obtain pertinent information on the outcome measures, and the effectiveness of the geriatric rehabilitation procedures. The study was conducted under the assumption, that the prevalence of depressive symptoms in the sample studied is within the globally recognized parameters and mainly affects older adults, living in nursing homes or without a life partner. Given the previously established impact of depressive symptoms in the population, it is expected that individuals with higher GDS scores (GDS ≥ 6) will have a poorer functional status and slower rehabilitation recovery. The results could provide important insights into the prevalence of depressive symptoms in older people and provide data for immediate interventions that improve recovery and the effectiveness of rehabilitation initiatives, potentially offering significant health and socio-economic benefits.

## 2. Materials and Methods

### 2.1. Participants

**Figure 1:**
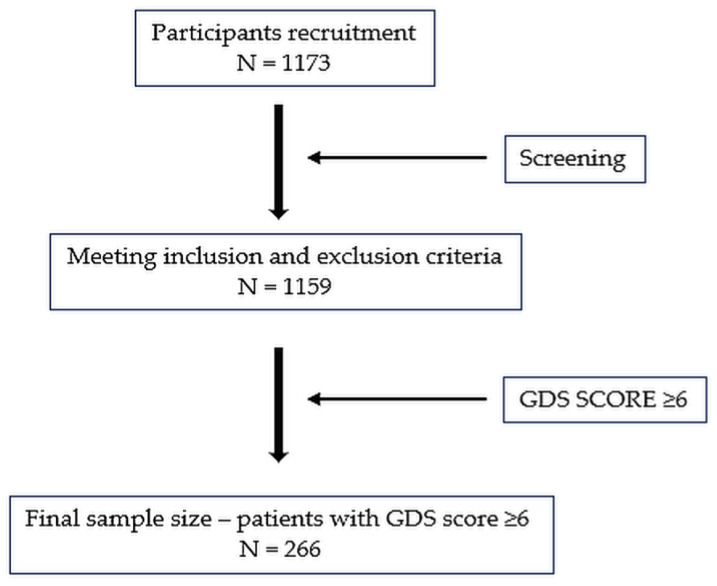
Flowchart of the number of participants at different stages in the study. N—number of participants.

The retrospective review of hospital records collected information on 1173 patients aged 65 years and older, who were admitted to the geriatric department of the Lucerne Cantonal Hospital in Wolhusen, Switzerland, between 2015 and 2020. Patients who took part in the study were in a stable state of health so they could participate without any immediate medical risks. A total of 1159 patients were included in the study, based on the exclusion criteria (Figure 1). Exclusion criteria included individuals younger than 65 years, patients with severe and uncontrolled health complications (e.g., advanced heart failure), patients with complete immobility making active rehabilitation impossible, patients with incomplete documentation, and individuals with cognitive impairments affecting their ability to follow instructions. Cognitive deficits were validated by a psychological assessment during hospitalization, with the resulting data documented in the hospital’s accessible medical records. Subjects were divided into four age cohorts: 65–70 years, 71–80 years, 81–90 years, and those older than 90 years.

### 2.2. Procedure and Materials

The presence of depressive symptoms was assessed using the standardized Geriatric Depression Scale (GDS) Short Form questionnaire, which was developed by Yesavage et al. [34]. The GDS has shown a 92% sensitivity and a specificity of 89% in detecting depressive symptoms, with excellent reliability (α = 0.91) [35,36]. The GDS short form consists of standardized 15 questions. Authors Sheikh and Yesavage validated both the long and short formats of the GDS (30 and 15 questions, respectively) in 1986. In a validation study, both iterations were found to be very effective in discriminating between individuals with and without depression, while showing a strong correlation [36]. The conclusion was that the short version of the GDS is suitable for screening for the presence of depressive symptoms. The American Psychological Association advocates using the GDS short questionnaire because it can be completed in approximately 5 to 7 min. This makes it particularly suitable for people who tire easily or cannot concentrate for long periods [37]. A GDS short-form questionnaire score of more than 5 requires further psychological assessment, as it indicates depressive states.

The Functional Independence Measure (FIM) assesses disabilities with different diagnoses. It was developed by a task force sponsored by the American Academy of Physical Medicine and Rehabilitation and the American Congress of Rehabilitation Medicine and published in 1987 by Keith, Granger, Hamilton, and Sherwin [38]. The FIM assesses six areas of functioning (self-care, sphincter control, transfers, locomotion, communication, and social cognition), which are divided into two domains (motor and cognitive). Each item is scored from 1 (complete support) to 7 (complete independence). The cumulative FIM score varies from 18 to 126, with a higher score indicating greater functional independence. The scores are categorized into four different levels. Complete independence (scores of 104–126) means that the person does not need help with any activity. Modified dependence is divided into two levels: Partial support needed (scores between 61 and 103 indicate that the person needs help with no more than 25% of tasks) and moderate support needed (scores between 19 and 60 indicate that the person needs help with up to 50% of tasks). Complete dependence is defined by a score of 18 points, which means that the person needs full support for all activities. The FIM is used to assess the patient’s level of disability and to monitor the changes in condition, in response to rehabilitative or medical interventions [39,40,41,42].

The Tinetti test, formally known as the Performance-Oriented Mobility Assessment, assesses a person’s perception of balance and stability during activities of daily living as well as their fear of falling. It is a credible measure for assessing the risk of falling. The Tinetti test consists of two different components: an assessment of gait and an assessment of the balance, on a 3-point ordinal scale (0, 1, and 2). A maximum total score of 28 can be achieved, with 12 points awarded for gait and 16 points for balance. Lower scores correlate with an increased risk of falling. A score of 18 indicates a high risk of falling, scores between 19 and 23 indicate a medium risk and a score of 24 indicates a lower risk of falling [43,44].

### 2.3. Statistical Analysis

The data were analyzed using TIBCO Statistica 13.3, a software specifically designed for the statistical analysis of datasets. The key socio-demographic characteristics of participants were evaluated through descriptive frequency analysis.

The Kolmogorov–Smirnov test was conducted to evaluate the variables’ distribution. Due to the non-normal distribution of the study variables, the prevalence of depressive symptoms across different age groups, genders, and marital statuses was analyzed using the χ^2^-test. Additionally, the Wilcoxon signed-rank test was used to assess the variance of FIM scores at admission and discharge. However, data related to the rehabilitation duration was found to be parametric, therefore, the *t*-test for independent samples was applied to analyze this variable. All statistical tests were conducted at a 95% confidence interval.

In addition, a correlation analysis was conducted to examine the relationships between FIM scores in individuals with and without depressive symptoms. Moreover, this analysis investigated the association between Tinetti test results obtained at admission and discharge. On the other hand, the examination of the effect sizes provided valuable insights, enabling us to draw well-founded conclusions about issues such as the differences in the prevalence of depressive symptoms between genders and the comparison of individuals living in nursing homes with those residing independently at home. This thorough and methodical approach can significantly enrich our understanding of depression across diverse demographic groups, emphasizing the need for targeted interventions.

## 3. Results

This study examines the prevalence of depressive symptoms and their profound impact on the functional status of patients currently undergoing geriatric rehabilitation, providing insights into the intersection of mental health and geriatric care. The key characteristics of the subjects involved in this research project are systematically listed in Table 1. A total of 1159 patients were included in this study, with an average age of 83.1 years, highlighting the participation of older people. Most participants were female (63.6%), while the proportion of male participants was 36.4%, highlighting the gender dynamics in this cohort. In addition, a considerable percentage of participants, 56.3%, were between 81 and 90 years old, indicating a striking concentration of older people in this age group.

Most of the respondents (84.2%) came to the geriatric ward from their residences, while a minority (15.8%) came from care facilities. A notable proportion of respondents, 47.7%, reported being widowed, highlighting the social dynamics in this population, closely followed by married individuals who made up 40.2% of the sample. On average, people in the sample participated in rehabilitation for 20.2 ± 7.1 days.

### 3.1. The Frequency of Depressive Symptoms in General in the Geriatric Population

A total of 266 respondents participated in the study, revealing a concerning 22.9% who were diagnosed with depressive symptoms (GDS ≥ 6). Among those affected, 177 (66.5%) were females, contrasted with 89 (33.5%) males. The demographic distribution clearly shows the profound impact of depressive symptoms across genders (d = 0.63), with statistically significant differences (χ^2^ = 29.11, *p* < 0.01) in their prevalence as detailed in Table 2. This evidence emphasizes the need to address the gender disparities in mental health in the observed sample.

**Figure 2:**
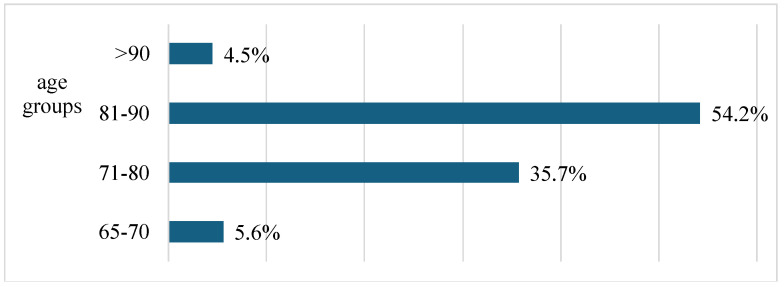
The percentages of participants with depressive symptoms in different age groups.

Moreover, the results obtained with the χ^2^-test demonstrate significant differences in the prevalence of depressive symptoms across different age groups (χ^2^ = 187.08, *p* < 0.01). Notably, the highest percentage of participants with depressive symptoms (54.2%) is found among those aged 81 to 90, whereas the lowest prevalence (4.5%) is observed in individuals over the age of 90, as illustrated in Figure 2.

However, it is crucial to emphasize that when comparing women and men in the different age cohorts, there was no statistically significant difference in the prevalence of depressive symptoms (χ^2^ = 0.05 *p* = 0.997), (Table 3).

At the same time, the analysis shows that 19.2% of people with depressive symptoms live at home, while 80.8% live in care facilities, as shown in Table 2. Obtained results suggest that depressive symptoms are significantly more common among individuals living in nursing homes than among those living independently at home (χ^2^ = 101.11, *p* < 0.01). Notably, the large effect size calculated (d = 0.82) highlights the urgent need for enhanced mental health support for vulnerable populations in care settings.

**Figure 3:**
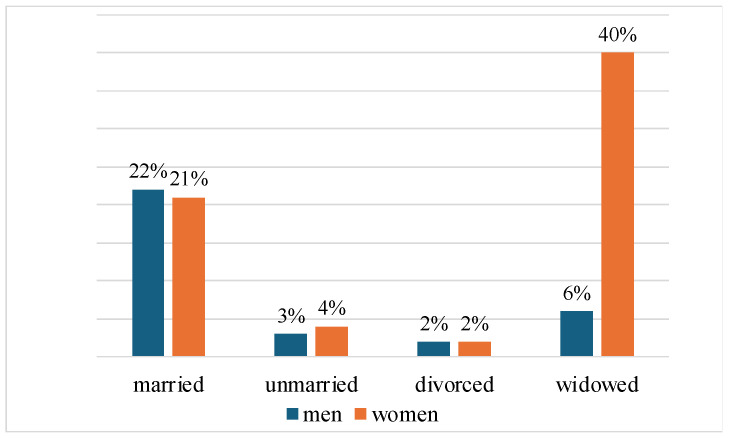
Percentage representation of participants with depressive symptoms regarding their gender and marital status.

In addition, depressive symptoms (GDS ≥ 6) occur in around a third, or 29%, of participants from care homes, while only 22% of people living in private households have similar symptoms. This illustrates the differences in mental health outcomes depending on living conditions. Further analysis clearly shows that there are no significant differences in the prevalence of depressive symptoms between the groups when the categories of respondents’ marital status are considered (χ^2^ = 160.29, *p* < 0.01), as shown in Table 2. However, when comparing men and women with GDS ≥ 6 according to their marital status (Figure 3), it becomes abundantly clear that the female cohort with depressive symptoms contains a significantly larger proportion of widows (40%), while the male cohort is predominantly characterized by married people (22%).

Furthermore, an analysis of the rehabilitation duration was conducted using a *t*-test for independent samples. This analysis compared the average rehabilitation times of 266 patients exhibiting depressive symptoms with those of 826 symptom-free patients, aiming to identify significant differences that could enhance patient care. Notably, the findings indicated that participants diagnosed with depressive symptoms required, on average, nearly two additional days of rehabilitation, compared to those without the symptoms, as shown in Table 4. However, with a calculated intermediate effect size (*p* = 0.012, d = 0.34) it is important to recognize that this two-day difference, while statistically significant, has a minimal impact on the overall rehabilitation process, suggesting that the additional time spent may not substantially alter recovery outcomes [45].

### 3.2. The Correlation Between Depressive Symptoms and Functional Status

**Figure 4:**
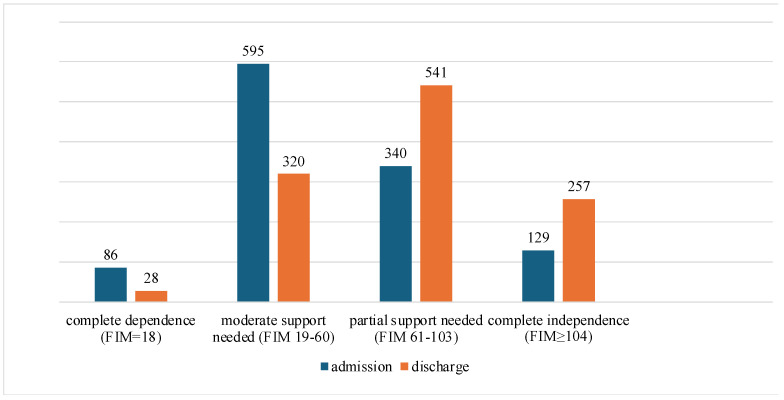
The number of participants on admission and discharge regarding the four levels of disability (complete dependence, moderate support needed, partial support needed, complete independence).

The study found no statistically significant correlation between FIM scores recorded at the time of patient admission and scores derived from the GDS. However, it is important to emphasize that a modest, but statistically significant correlation was found between these two important measures at the time of patient discharge, as indicated by the values of *p* = 0.043 and r = −0.06. This inverse correlation indicates that an improvement in functional status is associated with a decrease in GDS scores, which logically implies a corresponding decrease in the severity of depressive symptoms. Furthermore, the results from the non-parametric Wilcoxon matched-pairs test provide compelling evidence of significant changes in FIM scores when comparing admission scores to those at discharge across all FIM categories: complete dependence (*p* = 0.0003, d = 0.72), moderate support needed (*p* = 0.0001, d = 0.56), partial support needed (*p* = 0.0001, d = 0.56), complete independence (*p* = 0.0008, d = 0.60). Notably, there is a marked decrease in the number of patients classified as completely dependent and moderately dependent by the end of rehabilitation (see Figure 4). At the same time, there is a significant increase in patients classified as partially independent and fully independent, showing a clear and positive trend in these two categories in terms of improvement in functional status at discharge. However, the effect sizes calculated for each category indicate a moderate impact on the overall interpretation of these findings.

The findings of this study are compelling, revealing a lack of a significant relationship (*p* = 0.288, r = 0.117) between the FIM scores at admission and those at discharge for individual patients. Furthermore, almost no correlation (r = 0.023) was found between the FIM scores of cohorts that included individuals with GDS ≥ 6 and a GDS < 6. These results (d = 0.82) emphasize that the presence of depressive symptoms does not significantly influence the results of functional assessment measures, underscoring the need for further exploration in this area.

**Figure 5:**
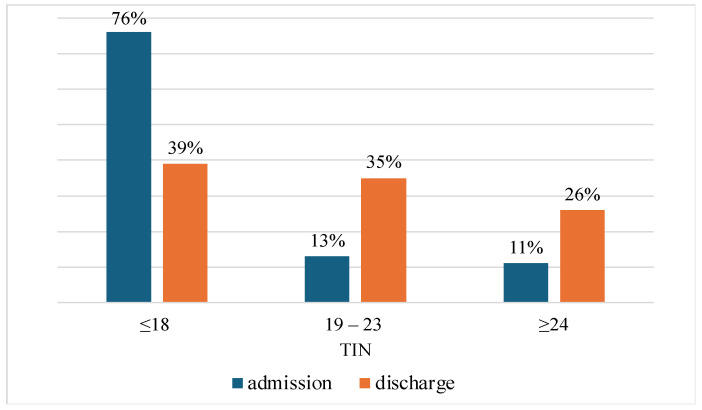
Percentage representation of patients on admission and discharge, related to Tinetti score categories.

The relationship between depressive symptoms and the Tinetti score was then examined in detail, as 76% of all participants were classified as at risk of falling. First, a statistically significant difference was found when Tinetti scores at the time of admission were compared with those at the time of discharge (χ^2^ = 5.04, *p* < 0.001). In addition, as can be seen in Figure 5, the proportion of patients who were categorized as having a “high fall risk” (TIN ≤ 18) decreased by a remarkable 37% after rehabilitation (76% to 39%).

Further analysis revealed that in the subgroup of participants who achieved a GDS score of 6 or higher (confidence interval: 6.46 to 6.74), there was no statistically significant correlation between the results of the mobility test conducted on admission (*p* = 0.835, r = 0.211) or at discharge (*p* = 0.336, r = 0.184) and the GDS score. This indicates that depressive symptoms do not have a measurable influence on the Tinetti score, which is an important tool for assessing the mobility of older adults.

When comparing individuals with a GDS ≥ 6 with those with a GDS < 6, there were no statistically significant differences in the results of the Tinetti mobility assessment (χ^2^ = 11.02, *p* = 0.275). Nonetheless, the calculated effect size (d = 0.34) suggests that the practical implications of these findings are limited, warranting further exploration into additional factors that may impact mobility within this demographic.

## 4. Discussion

Age is a central and highly influential aspect of human existence. The obstacles associated with aging are becoming increasingly important as society develops and technology advances. The unprecedented increase in the global population of older people will intensify in the coming decades, particularly in the developing countries [46].

Today’s society is faced with a complex challenge resulting from the increasing prevalence of physical and mental illnesses and the lengthening of life expectancy. The aging of the population is only one aspect of this problem, which is also characterized by lifestyle changes, environmental influences, and various other factors [46]. In our study, the average age of participants was 83.1 years, with 56.3% of participants in the 81 to 90 age group, which may lead to recall bias due to memory lapses, which could affect the accuracy of self-reported data. The vast majority of respondents (84.2%) were referred to the geriatric unit from home, while only 15.8% were referred to assisted living facilities. This scenario raises important questions about the support systems and overall quality of life of older people in their home environment, as well as the accessibility and standards of services provided in care facilities.

Depression is the second most common mental disorder in the elderly population. The World Health Organization estimates the overall prevalence of depression at 10–20%, a figure that is consistent with the results of our study, in which 266 (22.9%) of respondents were found to have depressive symptoms (GDS score ≥ 6). Numerous factors increase the risk of developing depressive symptoms in older adults. These include various acute and chronic illnesses, metabolic syndrome, and various mental disorders [47]. Of particular note is the socioeconomic status of older adults and adequate social support, as noted in studies by researchers such as Zhou et al. and Xue et al. [48,49]. Both mild and severe forms of depression, which are recognized as critical mental health problems in nursing home residents, affect up to 30% of this population [50,51]. A meta-analysis conducted by Zenebe et al. shows that the prevalence of depression in older people is higher in developing countries (40.78%) than in industrialized countries (17.05%) [1]. Globally, depression is more prevalent in older adults by 28.4%, with significant differences between studies, as shown in the meta-analyses by Hu et al. and Abdoli et al. [52,53]. These results emphasize the need to investigate the prevalence of depressive symptoms in the older population in different regional contexts, taking into account the geographical, cultural, and sociodemographic characteristics of specific areas. Although Switzerland is one of the countries with the highest GDP in the world, our study revealed a relatively high prevalence of depressive symptoms in older people. The results obtained indicate that depressive symptoms are significantly more common in people living in nursing homes than in people living independently at home (χ^2^ = 101.11, *p* < 0.01). The large effect size calculated (d = 0.82) highlights the urgent need for improved mental health support for vulnerable populations in care homes. Many residents face emotional and social loss when moving into a care home, which undoubtedly leads to feelings of isolation and a profound sense of the loss of control over their lives. In addition, older people’s mental well-being can be severely affected by changes in their environment, limited opportunities for active social participation, and reduced autonomy. Depressive symptoms in care home residents often go unrecognized and untreated, representing a significant failure of the healthcare system. Such untreated symptoms undeniably lead to a reduced quality of life, impaired physical functioning, increased rates of premature mortality, and higher rates of hospitalization. Therefore, formulating innovative strategies for caring for older people in nursing homes is essential. A notable initiative in this context is the interdisciplinary DAVOS project by Tesky et al. in Germany, which aims to implement an innovative, stepwise case management program to improve the treatment of depression in nursing home residents [50].

Although care homes may be an option for some older people, most prefer to live in their own homes. The importance of caring for and supporting older people in their home environment is undeniable. The findings presented by Lette and colleagues, drawn from a comprehensive review of thirteen different case studies, clearly highlight the need for integrated care services across Europe to prioritize the safety of older adults and implement a wide range of multifaceted strategies to achieve this compelling goal [54]. It is critical to recognize that deeper integration of health and social care solutions is essential to enhance older adults’ sense of safety and ensure a comprehensive and person-centered support system, that fully addresses their unique needs and concerns [54]. It is essential to ensure that older people receive the necessary psychosocial support and care in their home environment, including regular medical check-ups, therapeutic interventions, opportunities for social engagement, and opportunities for physical activity.

The modular framework provides tailored mental health support. It evaluates the effectiveness of the intervention in alleviating depression in this vulnerable population, as explained in the article by Reynolds et al. [55]. Emphasis must be placed on considering the different needs and challenges of individual residents and tailoring care approaches accordingly. These considerations include emotional support, access to activities and social interaction, and the promotion of autonomy and control over one’s life. The results obtained show significant differences in the prevalence of depressive symptoms between the different age groups (χ^2^ = 187.08, *p* < 0.01). A significant 54.2% of the study participants fell into the specific age group of 81- to 90-year-olds, supporting the assertion that the incidence and prevalence of depressive symptoms increase significantly with age and becomes more pronounced, although the lowest prevalence (4.5%) is observed in people over 90 years of age. However, as has been strongly asserted and reiterated, this fact may lead to recall bias due to inevitable memory lapses, which could significantly affect the reliability and accuracy of the data that participants self-report about their mental health status [56]. These data unequivocally underscore the need to recognize and support the older population. These measures become even more important in the context of people living independently.

Of the participants with a GDS ≥ 6, 177 (66.5%) were female, compared to 89 (33.5%) male participants. This demographic distribution highlights the significant impact of gender on the prevalence of the depressive symptoms (d = 0.63), with statistically significant differences (χ^2^ = 29.11, *p* < 0.01). This result is consistent with previous studies that have shown that women are more likely to develop depressive symptoms [47]. It is important to emphasize that when comparing women and men across different age cohorts, no statistically significant difference was found in the prevalence of depressive symptoms.

There are considerable differences in the prevalence of depressive symptoms depending on marital status. A stable and nurturing marital relationship undeniably promotes emotional support and a sense of belonging, which effectively reduces the risk of depression [57]. Our study clearly shows that the majority of people with depressive symptoms were women, particularly widows. The loss of a partner often leads to feelings of isolation and grief, which significantly increase the risk of developing depression. Previous research by Zhou et al. has clearly shown that middle-aged and older women are more prone to depression than their male counterparts and that middle-aged and older married individuals are significantly less likely to suffer from depression, compared to those who are separated, divorced, widowed, or never married [58]. Interestingly, our study revealed a higher prevalence of depressive symptoms in married men compared to single men. This finding may be attributed to several factors, including family expectations, marital and family stressors, economic challenges, and social and cultural norms that discourage men from expressing their emotional vulnerability or seeking help. In addition, these same factors may also contribute to the development of depressive symptoms in younger men. Married men inevitably bear the responsibility for their families, which often leads to feelings of overwhelm and diminished self-esteem, as they cope with the pressures of daily life. This phenomenon is particularly pronounced in older men, whose physical and mental health deteriorates faster than that of their female counterparts [59].

In this analysis, the average rehabilitation times of 266 patients with a GDS ≥ 6 were compared with those of patients with a GDS < 6, to identify significant differences that could improve patient care. The results showed that participants with higher GDS scores required almost two additional days of rehabilitation on average. However, with a calculated mean effect size (*p* = 0.012, d = 0.34), it should be noted that although this two-day difference is statistically significant, it only has a minimal effect on the overall rehabilitation process, suggesting that the additional time spent does not significantly affect recovery outcomes. Previous studies have shown that depression can hinder the progress of physical rehabilitation by reducing motivation and participation in therapy. However, concurrent therapeutic procedures have been shown to alleviate depressive symptoms [60,61]. At the same time, it must not be forgotten that any prolongation of the rehabilitation process has financial implications, which will undoubtedly be the focus of future research and analyses, comparing the cost-effectiveness of rehabilitation procedures. It is essential to include targeted mental health interventions in geriatric programs. By incorporating mandatory mental health assessments into rehabilitation practice and using validated screening tools, healthcare providers can improve the effectiveness of patient treatment and outcomes, while also bringing certain financial implications.

Although our examination of the data revealed no substantial or appreciable correlation between FIM scores recorded at admission and GDS scores obtained during the same initial assessment period, it is important to emphasize that a small but statistically significant correlation occurred at the time of discharge, as evidenced by the statistical values of *p* = 0.043 and r = −0.06. The existence of this negative correlation suggests that individuals with better functional status tend to have lower GDS scores; conversely, individuals with higher GDS scores are likely to show poorer functional status. However, it is important to recognize that due to its extremely weak correlation, this relationship is unlikely to have a clinically relevant impact on patient outcomes or treatment decisions. Allen et al. demonstrated a statistically significant relationship between changes in FIM scores and a reduction in depressive symptoms from admission to discharge; however, no such relationship was found from discharge to follow-up [62].

Patients who have suffered a stroke or a heart attack show significantly lower efficacy in functional recovery compared to patients without depressive symptoms. A study by Wada et al. and Sharma et al. has shown that patients with depression have a slower functional recovery after a stroke than patients without depression [63,64]. Studies in the field of cardiac rehabilitation emphasize the significant impact of depression on patient recovery. Depression has been shown to hinder the rehabilitation process, slowing progress and reducing overall functional outcomes [65,66,67]. Depression is a prevalent comorbidity among individuals with cancer, often leading to higher mortality rates. It also significantly diminishes the quality of life, complicating both treatment and recovery [68]. This is a clear indication that these patients need to be provided with enhanced psychological resources, including timely assessments and effective interventions.

The study did not find a statistically significant correlation between FIM scores at enrollment and GDS scores. However, it is important to note that a modest but statistically significant correlation was found between these two key scores at patient discharge, with values of *p* = 0.043 and r = −0.06. This inverse relationship suggests that improvements in functional status are associated with a decrease in GDS scores, which is logically associated with a decrease in the severity of depressive symptoms. This result is in line with some previous studies, such as Nyunt et al. [69]. In addition, the non-parametric Wilcoxon matched-pairs test revealed significant changes in FIM scores when comparing admission and discharge scores in all FIM categories: complete dependence (*p* = 0.0003, d = 0.72), moderate need for support (*p* = 0.0001, d = 0.56), partial need for support (*p* = 0.0001, d = 0.56) and complete independence (*p* = 0.0008, d = 0.60). It is noteworthy that the number of patients who were classified as completely or moderately dependent fell significantly at the end of rehabilitation. Conversely, the number of patients classified as partially or fully independent increased significantly, indicating a clear, positive trend toward functional improvement at discharge. However, the effect sizes for the individual categories indicate a moderate impact on the overall interpretation of these results. The results of the study also show that there is no significant correlation (*p* = 0.288, r = 0.117) between the FIM scores on admission and discharge of individual patients. In addition, almost no correlation (r = 0.023) was found between the FIM scores of cohorts with a GDS ≥ 6 and those with a GDS < 6. These results (d = 0.82) strongly suggest that the presence of depressive symptoms does not significantly influence the results of functional assessments, highlighting the need for further research in this area. Rehabilitation protocols must be improved, especially for individuals with impaired functional status who often have more acute disease manifestations. These rehabilitation protocols must be carefully tailored to each individual’s specific needs and abilities and include a holistic strategy that encompasses the physical, psychological, and social dimensions of health. For individuals with low functional status, it is essential to modify and expand the rehabilitation program to achieve optimal results. A systematic increase in training intensity, individualized therapeutic interventions, and assistance with daily activities are essential to achieve maximum functional independence. After discharge, individuals with limited functional status are often transferred to temporary or permanent care facilities.

The effectiveness of rehabilitation interventions for older people with a higher functional status on admission, which continues to improve during hospitalization, has been proven. People with a higher functional status generally have greater autonomy and competence in performing everyday tasks, which significantly enhances self-esteem and self-actualization, as Muszalik et al. found [70]. In contrast, impaired functional status severely limits a person’s ability to perform basic activities, causing feelings of powerlessness and frustration and thus increasing the risk of depressive states. To ensure appropriate care after discharge, it is crucial to thoroughly assess the functional status of elderly patients before they return home. Necessary adjustments to the living environment, provision of medical equipment or therapeutic interventions, and assistance with daily tasks must be made. In addition, emotional support and education are critical to ensure that individuals have the necessary resources and skills to manage the transitions and challenges associated with returning home. With a comprehensive approach, that takes into account the functional status, psychological well-being, and specific needs of each older person, exemplary care can and must be provided after discharge from the hospital to improve overall well-being and clinical outcomes, minimize distress, and enhance the quality of life [71,72].

The correlation between depressive symptoms and the Tinetti score was investigated, as 76% of participants were classified as being at an increased risk of falling. A significant difference in the Tinetti score was found between admission and discharge (*p* < 0.001), but with no difference between patients with a GDS ≥ 6 and a GDS < 6. Fall prevention is critical in elderly care as falls are very common, cause serious injuries, and affect quality of life. Effective fall prevention, which includes risk assessments, environmental adjustments, physical activity, education, and medical interventions, is essential for improving patient safety and quality of life in geriatric rehabilitation.

### 4.1. Limitations of the Study

This study used a retrospective cohort design, which undeniably introduces bias in data collection and participant selection. The reliance on historical data certainly affects the accuracy of the results. Conducting the study in a single hospital in Switzerland significantly limits the generalizability of the results to other populations or settings, particularly in different cultural or economic contexts. The specific assessment tools such as the GDS and the FIM, although validated, do not capture the full spectrum of depressive symptoms or functional abilities. While it is undeniable that the GDS short form has a significant level of sensitivity and specificity, that is commendable in the realm of diagnostic tools, it is important to recognize that this tool alone should not be considered a definitive diagnostic solution. It is therefore strongly recommended that individuals undergo a much more comprehensive professional assessment to ensure accurate and reliable results. The study’s failure to follow participants longitudinally is essential for assessing the long-term outcomes of rehabilitation and the lasting impact of depressive symptoms. In addition, differences in rehabilitation protocols and support offered to participants could have significantly affected the outcomes.

### 4.2. Practical Implications

The study shows a high rate of depressive symptoms in older adults undergoing rehabilitation in Switzerland and underlines the urgent need to address mental health in geriatric care. Better screenings, with reliable tools such as the GDS, are essential to detect and treat depressive symptoms early, which can improve patient recovery. In addition to measuring prevalence, this study also examines the impact of depressive symptoms on functional abilities. This emphasizes that mental health is an important component of effective physical rehabilitation. The results show that individuals with depressive symptoms require a longer rehabilitation period, providing important insights for healthcare providers to improve the efficiency of rehabilitation. The prolonged rehabilitation time represents an additional economic burden, highlighting the need for healthcare systems to allocate resources to mental health. Globally, countries, particularly those with aging populations, will increasingly face depression in older adults, especially in the developing regions. Global health initiatives should prioritize culturally sensitive research into the prevalence and impact of depression, to develop effective intervention strategies. The integration of social services into health care is essential to provide comprehensive support for older adults.

## 5. Conclusions

The results of this study highlight the significant impact of depressive symptoms on the various aspects of rehabilitation in older adults. Depressive symptoms were more common in women, particularly widowed women, and were also more common in older adults living in care facilities. Although the presence of depressive symptoms had no significant effect on functional outcomes, there was a modest inverse correlation between functional improvements and a decrease in the severity of depressive symptoms. Remarkably, depressive symptoms did not appear to have a significant effect on mobility, as measured by the Tinetti score, or on the rehabilitation duration, although rehabilitation time increased slightly. This highlights the need for further research on the relationship between depression and rehabilitation, particularly with a focus on improving the mental health support in geriatric rehabilitation facilities. In addition, the findings point to the importance of considering the financial implications of increased rehabilitation time and the potential benefits of including mental health support interventions in rehabilitation programs for older people.

## Figures and Tables

**Table 1:** Basic sociodemographic characteristics of participants.

Characteristics	N	%
Gender		
women	737	63.6
men	422	36.4
**Age**		
65–70	48	4.2
71–80	322	27.8
81–90	653	56.3
>90	136	11.7
**Average age**	83.1	
**Habitation**		
home environment	976	84.2
institution (nursing home)	183	15.8
**Marital status**		
married	466	40.2
divorced	60	5.2
unmarried	80	6.9
widowed	553	47.7

N—number of participants, %—the percentage of participants.

**Table 2:** Sociodemographic characteristics of participants with depressive symptoms and the results of a statistical analysis of the comparison of the frequency of depressive symptoms to their gender, age, habitation, and marital status.

Characteristics	N	%	*p*	χ^2^	d
Gender					
women	177	66.5	<0.01	29.11	0.63
men	89	33.5			
**Age (years)**					
65–70	15	5.6	<0.01	187.08	
71–80	95	35.7			
81–90	144	54.2			
>90	12	4.5			
**Habitation**					
institution (nursing home)	215	80.8	<0.01	101.11	0.82
home environment	51	19.2			
**Marital status**					
married	115	43.2	<0.01	160.29	
divorced	11	4.1			
unmarried	19	7.1			
widowed	121	45.6			

N–number of participants, %—the percentage of participants, *p*—*p*-value, χ^2^—chi-square value, d—effect size.

**Table 3:** Comparison of prevalence of depressive symptoms between men and women by age groups.

Age (Years)	M	W	χ^2^	*p*
65–70	5	10	0.05	0.997
71–80	31	64		
81–90	49	95		
>90	4	8		

M—number of male participants, W—number of female participants, χ^2^—chi-square value, *p*—*p*-value.

**Table 4:** Comparison of the duration of rehabilitation in groups of participants with and without depressive symptoms.

Variable	N	N (<6)	N (≥6)	Mean < 6	Mean ≥ 6	*p*	d
duration of rehabilitation (days)	1159	893	266	19.87	21.70	0.012	0.34

N—number of participants, Mean—mean value, *p*—*p*-value, d—effect size.

## Data Availability

The datasets generated and analyzed during the current study are available from the corresponding author upon reasonable request.

## Abbreviations

The following abbreviations are used in this manuscript:

GDS	Geriatric Depression Scale
FIM	Functional Independence Measure
GDP	Gross domestic product
